# Supplementary material for: Zero-phase-delay synchrony between interacting neural populations: implications for functional connectivity-derived biomarkers
Source: Imaging Neurosci (Camb). 2025 Nov 10;3:IMAG.a.985. doi: 10.1162/IMAG.a.985 (PMC12603661; doi:10.1162/IMAG.a.985)
Supplement: Supplementary Material [file IMAG.a.985_Supp.docx]

# Supplementary Materials

**Supplementary Methods: Investigating phase-delay distributions due to artefact**

To investigate if the phase-delay distributions in Fig. 1 could be driven by signal leakage and/or volume conduction (‘common-mode’) artefact, we simulated artefact in randomly generated, uncorrelated timeseries with 50 regions of interest. For each pair of simulated signals, $s_{i}(t)$ and $s_{j}(t)$, we added a linear component of $s_{j}(t)$ to $s_{i}(t)$. The extent of artefact was toggled between a ‘common-mode factor’ of between 0.0 and 1.0.

Next, we investigated whether the underlying neurobiology of interacting region-pairs – specifically, homotopic versus non-homotopic pairs – significantly affected the phase-delay distribution when common-mode artefact was likely constant. Homotopic region-pairs were chosen as they are established to have a unique phase-delay distribution (Engel et al., 1991; O’Reilly & Elsabbagh, 2021). To ensure comparable levels of common-mode artefact between groups, we selected a matched set of non-homotopic region-pairs with similar Euclidean distance properties. This was achieved by identifying 34 non-homotopic pairs (from 10,000 random combinations) whose median and interquartile range of Euclidean distance most closely matched those of the 34 homotopic pairs. Finally, we compared the proportion of near-zero-phase-delay connectivity in homotopic and non-homotopic region-pairs using the Chi-Squared test.

**Supplementary Results**

Although the trimodal distributions of phase-delays seen in Fig. 1b-d are consistent with invasive recordings (Engel et al., 1991; O’Reilly & Elsabbagh, 2021), the presence of signal leakage and/or volume conduction (‘common-mode’ artefact) in signals is also sufficient to create a trimodal phase-delay distribution. This is illustrated in Supplementary Figure 1 using randomly generated, filtered signals from uncorrelated sources. The phase-delay distributions of these random timeseries adopt a more prominent trimodal pattern as the extent of common-mode artefact between region-pairs increases. As there may be residual common-mode artefact even in region-pairs > 80mm apart or between homotopic region-pairs, we investigated if this was driving the phase-delay distributions in Fig 1c-d.

The extent of common-mode artefact in source reconstructed data is a function of the Euclidean distance between region-pairs (Fig. 1a and Bastos & Schoffelen, 2016; Gohel et al., 2017; He et al., 2019; Hipp et al., 2012). Therefore, if the phase-delay distributions in Fig. 1c-d are predominantly driven by such artefact, their phase-delay distributions should *not* differ from a sample of regions matched on Euclidean distance distribution, irrespective of underlying neurobiology. To evaluate this, we leveraged the unique phase-delay distribution between homotopic region-pairs. Specifically, we compared the phase-delay distribution of the 34 homotopic region-pairs (median Euclidean distance = 57mm, IQR = 59mm) with 34 non-homotopic interhemispheric region-pairs matched on Euclidean distance (median = 57mm, IQR = 58mm). Across all frequency bands, we found that the percentage of near-zero-phase-delay connectivity was significantly different between the sets of region-pairs (e.g. in alpha: homotopic region-pairs = 83%, non-homotopic region-pairs matched on distance = 56% (*X*^2^ (1, n = 5,202) = 15.9, *p* < .001)). Therefore, the phase-delay-distributions are significantly influenced by the underlying neurobiology of sources at this Euclidean distance distribution. Given that common-mode artefact does not dominate the phase-delay distribution at this Euclidean distance distribution, and that the impact of this artefact decreases with distance, we can extrapolate our findings to region-pairs > 60mm.

**Supplementary Fig. 1: Effect of signal leakage / volume conduction (‘common-mode’) artefact on phase-delay distributions**


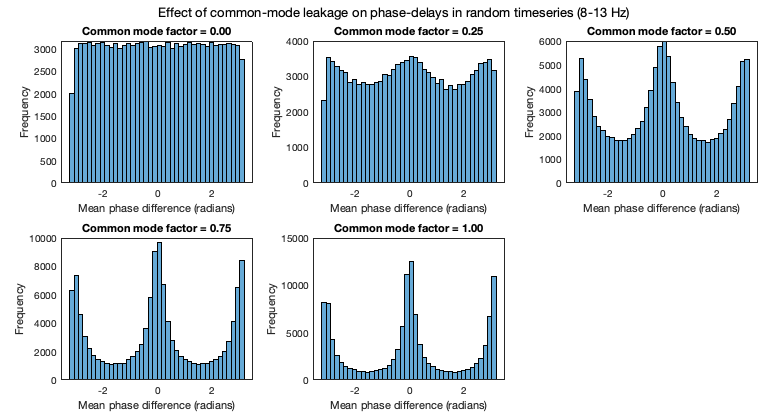


b


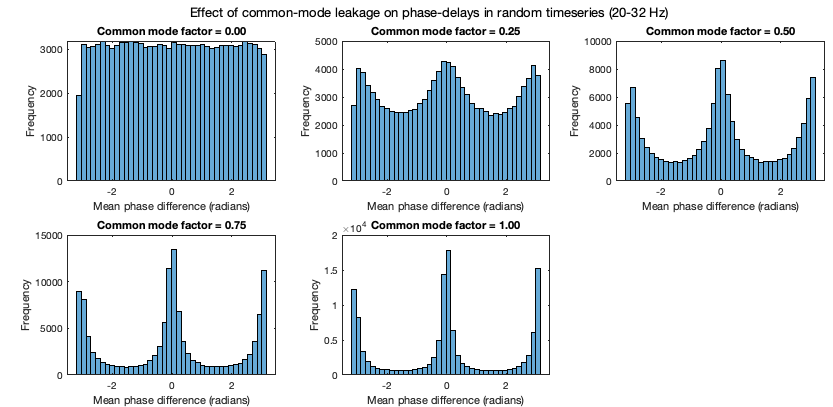


Effect of signal leakage / volume conduction (‘common-mode’) artefact on phase-delay distributions in randomly generated, filtered signals from uncorrelated sources, in the **a)** alpha and **b)** high-beta bands. The phase-delay distributions of random timeseries adopt a more prominent trimodal pattern as the extent of common-mode artefact between region-pairs increases.

| **Supplementary Table S1 – Comparing the percentage of near-zero-phase-delay functional connectivity between homotopic interhemispheric, heterotopic interhemispheric and non-homotopic region-pairs with a matched distance distribution as homotopic pairs.** df = degrees of freedom. *** p < .001, ** p < .01 | | | | | |
| --- | --- | --- | --- | --- | --- |
|  | Percentage of near-zero-phase-delay connectivity between region-pairs | | | | |
| Freq (Hz) | Homotopic interhemispheric  (%) | Heterotopic interhemispheric | | Non-homotopic region-pairs with matched distance distribution to homotopic interhemispheric | |
|  |  | (%) | X^2^ (df =1) | (%) | X^2^ (df = 1) |
| 1 – 4 | 84 | 55*** | 18.5 | 59*** | 14.1 |
| 4 – 8 | 86 | 58*** | 18.1 | 62*** | 13.7 |
| 8 – 13 | 83 | 50*** | 23.0 | 56*** | 15.9 |
| 13 – 20 | 87 | 61*** | 16.2 | 66*** | 11.1 |
| 20 – 32 | 89 | 67*** | 12.9 | 71** | 9.0 |

| **Supplementary Table S2 - Intrasession edgewise absolute agreement of EEG functional connectivity methods.** Median edgewise absolute agreement was calculated for each edge, using the interclass correlation coefficient (2,1; ICC). n = 99. | | | | | | | |
| --- | --- | --- | --- | --- | --- | --- | --- |
| **Method** | **Freq (Hz)** | **Median ICC** | **IQR** | **Method** | **Freq (Hz)** | **Median ICC** | **IQR** |
| Coherence | 1 - 4 | .15 | .26 | Imaginary part  of Coherency | 1 - 4 | .02 | .16 |
|  | 4 - 8 | .19 | .30 |  | 4 - 8 | .03 | .15 |
|  | 8 - 13 | .20 | .30 |  | 8 - 13 | .07 | .16 |
|  | 13 - 20 | .24 | .31 |  | 13 - 20 | .03 | .16 |
|  | 20 - 32 | .31 | .36 |  | 20 - 32 | .07 | .18 |
| PLV | 1 - 4 | .69 | .29 | wPLI | 1 - 4 | -.01 | .13 |
|  | 4 - 8 | .75 | .28 |  | 4 - 8 | .02 | .14 |
|  | 8 - 13 | .71 | .31 |  | 8 - 13 | .17 | .18 |
|  | 13 - 20 | .84 | .20 |  | 13 - 20 | .04 | .15 |
|  | 20 - 32 | .88 | .14 |  | 20 - 32 | .09 | .17 |
| AEC | 1 - 4 | .37 | .43 | Orth  AEC | 1 - 4 | .07 | .15 |
|  | 4 - 8 | .47 | .45 |  | 4 - 8 | .10 | .18 |
|  | 8 - 13 | .48 | .42 |  | 8 - 13 | .14 | .20 |
|  | 13 - 20 | .57 | .42 |  | 13 - 20 | .16 | .18 |
|  | 20 - 32 | .71 | .36 |  | 20 - 32 | .04 | .15 |

| **Supplementary Table S3 – Intrasession consistency of the EEG functional connectivity adjacency matrix**. Zero-phase-inclusive methods had moderate to high consistency values, while zero-phase-exclusive methods had near 0 consistency values. | | | | | | | |
| --- | --- | --- | --- | --- | --- | --- | --- |
| **Method** | **Freq (Hz)** | **Median Consistency** | **IQR** | **Method** | **Freq (Hz)** | **Median Consistency** | **IQR** |
| Coherence | 1 - 4 | .34 | .16 | Imaginary part  of Coherency | 1 - 4 | .07 | .09 |
|  | 4 - 8 | .40 | .18 |  | 4 - 8 | .10 | .11 |
|  | 8 - 13 | .40 | .16 |  | 8 - 13 | .12 | .11 |
|  | 13 - 20 | .40 | .16 |  | 13 - 20 | .11 | .12 |
|  | 20 - 32 | .47 | .18 |  | 20 - 32 | .16 | .16 |
| PLV | 1 - 4 | .80 | .10 | wPLI | 1 - 4 | -.01 | .07 |
|  | 4 - 8 | .84 | .09 |  | 4 - 8 | .01 | .11 |
|  | 8 - 13 | .82 | .09 |  | 8 - 13 | .12 | .16 |
|  | 13 - 20 | .88 | .06 |  | 13 - 20 | .02 | .08 |
|  | 20 - 32 | .91 | .05 |  | 20 - 32 | .02 | .08 |
| AEC | 1 - 4 | .55 | .15 | Orth  AEC | 1 - 4 | .01 | .05 |
|  | 4 - 8 | .64 | .18 |  | 4 - 8 | .02 | .07 |
|  | 8 - 13 | .60 | .15 |  | 8 - 13 | .04 | .13 |
|  | 13 - 20 | .66 | .14 |  | 13 - 20 | .02 | .10 |
|  | 20 - 32 | .71 | .13 |  | 20 - 32 | .01 | .06 |

| **Supplementary Table S4 - Structural-function concordance findings are replicated** when analysing the **a)** KCL (n = 28) and **b)** Mannheim (n = 22) study sites separately. Across all frequency bands, concordance was significantly greater than 0 with zero-phase-inclusive methods (one-sided t-test or Wilcoxon sum-ranked test, *μ* = 0, *p* < .0001, Bonferroni corrected). Concordance was not significantly different from 0 for wPLI and orthogonalized AEC. Surprisingly, concordance was significantly less than zero for the imaginary part of coherency. Concordance for each zero-phase inclusive method was significantly higher than that for its methodologically corresponding zero-phase exclusive method (e.g. coherence versus imaginary part of coherency in 1-4 Hz), for each frequency band (paired samples t-test or Wilcoxon signed-rank test, *p* < .0001, Bonferroni corrected). *** concordance significantly different from zero, *p* < .0001, Bonferroni corrected. ^w^ = data not normally distributed, therefore one-sided Wilcoxon sign-rank test performed, otherwise one-sided t-tests performed. SFC = structure-function concordance; SD = standard deviation.  **4a) SFC at KCL (n = 28)** | | | | | | | |  |
| --- | --- | --- | --- | --- | --- | --- | --- | --- |
| **Method** | **Freq (Hz)** | **Mean SFC** | **SD** | **Method** | **Freq (Hz)** | **Mean SFC** | **SD** |  |
|  |  |  |  |  |  |  |  |  |
| Coherence | 1 - 4^w^ | .112*** | .049 | Imaginary part  of Coherency | 1 - 4^w^ | -.041*** | .041 |  |
|  | 4 - 8 | .119*** | .043 |  | 4 - 8 | -.075*** | .042 |  |
|  | 8 - 13 | .112*** | .035 |  | 8 - 13 | -.057*** | .045 |  |
|  | 13 - 20 | .116*** | .053 |  | 13 - 20 | -.066*** | .060 |  |
|  | 20 - 32 | .124*** | .050 |  | 20 - 32 | -.081*** | .041 |  |
| PLV | 1 - 4 | .142*** | .040 | wPLI | 1 - 4 | .000 | .049 |  |
|  | 4 - 8^w^ | .142*** | .039 |  | 4 - 8 | -.004 | .040 |  |
|  | 8 - 13^w^ | .132*** | .030 |  | 8 - 13 | -.013 | .040 |  |
|  | 13 - 20 | .144*** | .034 |  | 13 - 20 | -.007 | .033 |  |
|  | 20 - 32 | .146*** | .040 |  | 20 - 32 | -.007 | .045 |  |
| AEC | 1 - 4^w^ | .137*** | .046 | Orthogonalized AEC | 1 - 4 | .007 | .040 |  |
|  | 4 - 8 | .133*** | .038 |  | 4 - 8 | .006 | .035 |  |
|  | 8 - 13 | .120*** | .047 |  | 8 - 13 | .001 | .049 |  |
|  | 13 - 20 | .140*** | .036 |  | 13 - 20 | .006 | .039 |  |
|  | 20 - 32 | .141*** | .039 |  | 20 - 32 | .000 | .037 |  |

**4b) SFC at Mannheim (n = 22)**

| **Method** | **Freq (Hz)** | **Mean SFC** | **SD** | **Method** | **Freq (Hz)** | **Mean SFC** | **SD** |  |
| --- | --- | --- | --- | --- | --- | --- | --- | --- |
|  |  |  |  |  |  |  |  |  |
| Coherence | 1 - 4 | .080*** | .027 | Imaginary part  of Coherency | 1 - 4 | -.034*** | .035 |  |
|  | 4 - 8 | .094*** | .032 |  | 4 - 8 | -.043*** | .049 |  |
|  | 8 - 13 | .096*** | .043 |  | 8 - 13 | -.037*** | .052 |  |
|  | 13 - 20 | .094*** | .028 |  | 13 - 20 | -.040*** | .033 |  |
|  | 20 - 32 | .099*** | .041 |  | 20 - 32 | -.061*** | .054 |  |
| PLV | 1 - 4 | .112*** | .028 | wPLI | 1 - 4 | -.003 | .030 |  |
|  | 4 - 8 | .109*** | .025 |  | 4 - 8 | -.018 | .039 |  |
|  | 8 - 13 | .102*** | .029 |  | 8 - 13 | -.007 | .038 |  |
|  | 13 - 20 | .113*** | .024 |  | 13 - 20 | .013 | .030 |  |
|  | 20 - 32 | .115*** | .027 |  | 20 - 32 | .012 | .042 |  |
| AEC | 1 - 4 | .104*** | .029 | Orth AEC | 1 - 4 | .004 | .030 |  |
|  | 4 - 8 | .109*** | .030 |  | 4 - 8 | .015 | .038 |  |
|  | 8 - 13 | .100*** | .034 |  | 8 - 13 | .012 | .040 |  |
|  | 13 - 20 | .108*** | .028 |  | 13 - 20 | .012 | .031 |  |
|  | 20 - 32 | .114*** | .030 |  | 20 - 32 | .011 | .030 |  |

| **Supplementary Table S5 – Comparison of mean functional connectivity strength between homotopic region-pairs versus all other region-pairs when using the imaginary part of coherency.** Statistics calculated using the Wilcoxon rank sum test, *p* values Bonferroni corrected. | | | | | |
| --- | --- | --- | --- | --- | --- |
|  | Mean strength between region-pairs with imaginary part of coherency | | Statistics | | |
| Freq (Hz) | Homotopic | Non-homotopic | Z | T | *p* |
| 1 – 4 | .097 | .137 | 6.63 | 2582291 | < .0001 |
| 4 – 8 | .103 | .146 | 7.19 | 2584398 | < .0001 |
| 8 – 13 | .112 | .157 | 7.52 | 2585675 | < .0001 |
| 13 – 20 | .119 | .167 | 6.65 | 2582369 | < .0001 |
| 20 – 32 | .125 | .177 | 7.64 | 2586111 | < .0001 |


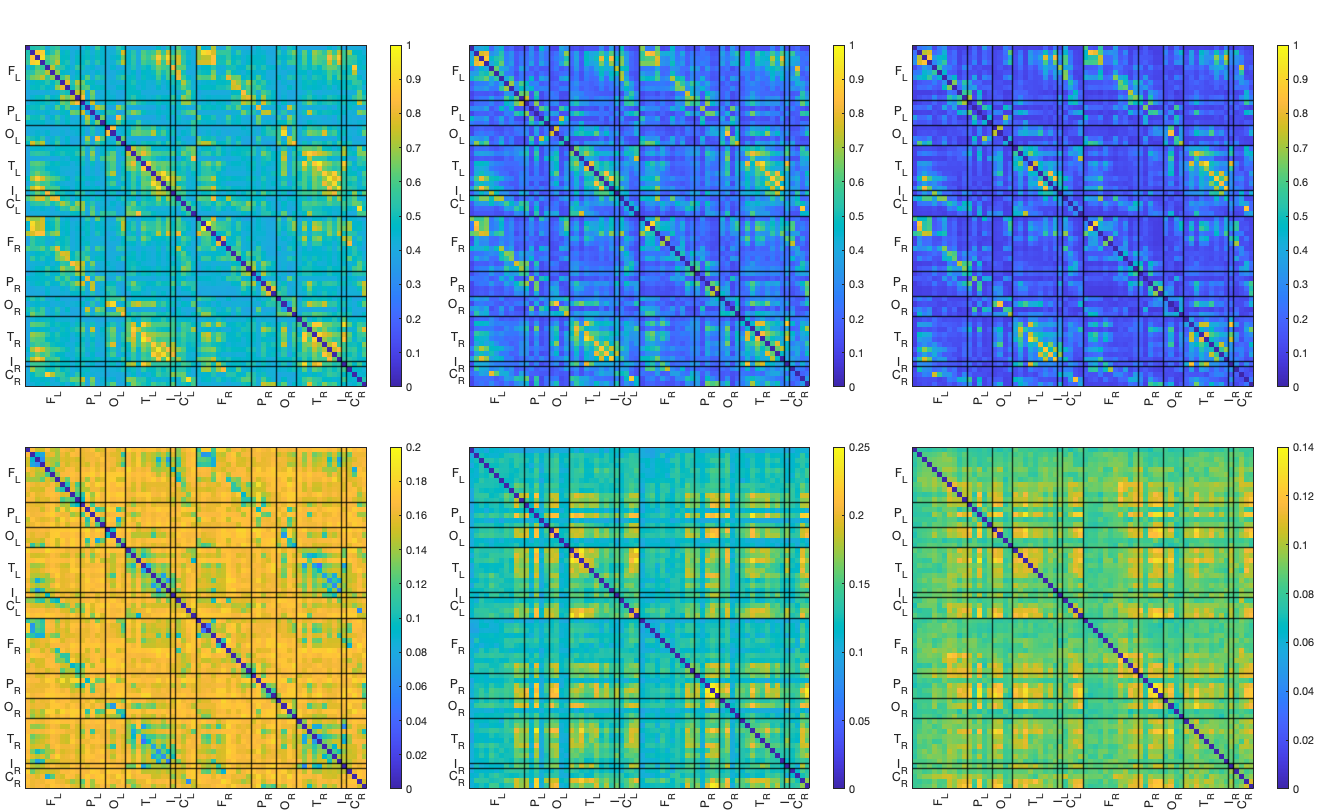


COH

PLV

AEC

Img COH

wPLI

Orth AEC


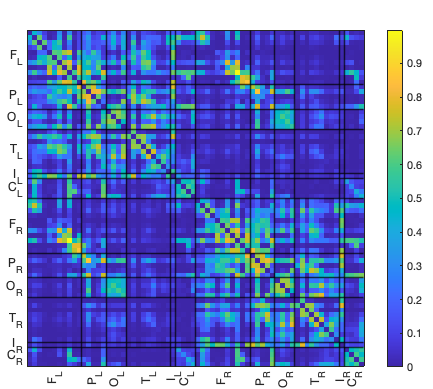


**b) Structural Connectivity**

**Supplementary Fig. 2: a) Edgewise functional connectivity in 8-13 Hz averaged across participants**

Mean edgewise **a)** EEG functional connectivity matrices (n = 153) in 8-13 Hz band and **b)** structural connectivity matrix (n = 50). Strong homotopic interhemispheric connectivity (seen at the off-diagonal edges in each adjacency matrix) was found with structural connectivity and with all zero-phase-inclusive functional connectivity methods. No zero-phase-exclusive methods showed prominent callosal connectivity. The imaginary part of coherency showed significantly weaker connectivity between homotopic interhemispheric region-pairs than between other region-pairs. Colour bars reflect connectivity strength; yellower colours denote higher connectivity strength. Brain regions in matrices are ordered by lobe and their proximity to each other. The axes are labelled with the lobe regions are found in: F = frontal, P = parietal, O = occipital, T = temporal, I = insular, C = cingulate. _L_ = left hemisphere. _R_ = right hemisphere.

| **c)** The order of ROIs in a) and b) from top to bottom and left to right | |
| --- | --- |
| **Region of interest (ROI)** | **Lobe** |
| Frontal Pole | Frontal |
| Medial Orbitofrontal | Frontal |
| Lateral Orbitofrontal | Frontal |
| Pars Orbitalis | Frontal |
| Pars Opercularis | Frontal |
| Pars Triangularis | Frontal |
| Rostral Middle Frontal | Frontal |
| Caudal Middle Frontal | Frontal |
| Superior Frontal | Frontal |
| Paracentral | Frontal |
| Precentral | Frontal |
| Postcentral | Parietal |
| Superior Parietal | Parietal |
| Supramarginal | Parietal |
| Inferior Parietal | Parietal |
| Precuneus | Parietal |
| Pericalcarine | Occipital |
| Lingual | Occipital |
| Cuneus | Occipital |
| Lateral Occipital | Occipital |
| Transverse Temporal | Temporal |
| Superior Temporal | Temporal |
| Banks STS | Temporal |
| Middle Temporal | Temporal |
| Inferior Temporal | Temporal |
| Fusiform | Temporal |
| Entorhinal | Temporal |
| Parahippocampal | Temporal |
| Temporal Pole | Temporal |
| Insula | Insular |
| Rostral Anterior Cingulate | Cingulate |
| Caudal Anterior Cingulate | Cingulate |
| Posterior Cingulate | Cingulate |
| Isthmus Cingulate | Cingulate |

| **Supplementary Table S6** **–** General linear model illustrating a significant age x pathlength interaction in predicting longitudinal changes in spatial working memory errors, when using the imaginary part of coherency. Participants aged 6-31-years. | | | | |
| --- | --- | --- | --- | --- |
|  | Est. coeff | Std. Error | t | *p* |
| Intercept | -223.60 | 118.10 | -1.89 | .06 |
| Pathlength (Img COH) | 229.10 | 119.10 | 1.92 | .06 |
| Age | 15.42 | 6.54 | 2.36 | .02 |
| Time 1 spatial working memory errors | -0.60 | 0.08 | -7.35 | .00 |
| T2-T1 time interval | 0.01 | 0.01 | 1.21 | .23 |
| **Pathlength (Img COH) x age** | **-15.80** | **6.58** | **-2.40** | **.02** |
| Model: *Longitudinal changes in spatial working memory errors ~ Pathlength (derived from imaginary part of coherency) + Age + time 1 spatial working memory errors + T2-T1 time interval + Pathlength (derived from imaginary part of coherency) x Age*. R^2^ = .39, R^2^_adj_ = .36, F_5/102_ = 12.9, *p* < .001. | | | | |

| **Supplementary Table S7** **–** General linear model illustrating a significant age x pathlength interaction in predicting longitudinal changes in spatial working memory errors, when using coherence. Participants aged 6-31-years | | | | |
| --- | --- | --- | --- | --- |
|  | Est. coeff | Std. Error | t | *p* |
| Intercept | -348.90 | 135.00 | -2.59 | .01 |
| Pathlength (COH) | 331.80 | 126.90 | 2.62 | .01 |
| Age | 20.36 | 7.23 | 2.82 | .01 |
| T2-T1 time interval | 0.01 | 0.01 | 1.45 | .15 |
| Time 1 spatial working memory errors | -0.59 | 0.08 | -7.04 | .00 |
| **Pathlength (COH) x age** | **-19.48** | **6.82** | **-2.86** | **.01** |
| Model: *Longitudinal changes in spatial working memory errors ~ Pathlength (derived from coherence) + Age + time 1 spatial working memory errors + T2-T1 time interval + Pathlength (derived from coherence) x Age*. R^2^ = .40, R^2^_adj_ = .36, F_5/102_ = 13.3, *p* < .001. | | | | |

| **Supplementary Table S8** **–** General linear model predicting longitudinal changes in spatial working memory ability in 6-12-year-olds using coherence. | | | | |
| --- | --- | --- | --- | --- |
|  | β | Std. Error | t | *p* |
| Intercept | 21.1 | 9.9 | 2.1 | .04 |
| **Pathlength (COH), standardised** | **3.8** | **1.5** | **2.62** | **.01** |
| Age | -2.05 | 0.83 | -2.46 | .02 |
| Time 1 spatial working memory errors | -0.25 | 0.17 | -1.50 | .14 |
| Model: *Longitudinal changes in spatial working memory errors ~ Pathlength (derived from coherence, standardised) + Age + time 1 spatial working memory errors*. R^2^ = .30, R^2^_adj_ = .23, F_3/28_ = 4.0, *p* = .017. | | | | |

| **Supplementary Table S9** **–** General linear model predicting longitudinal changes in spatial working memory ability in 18-31-year-olds using PLV. | | | | |
| --- | --- | --- | --- | --- |
|  | β | Std. Error | t | *p* |
| Intercept | -5.6 | 7.8 | -0.73 | .47 |
| **Pathlength (PLV), standardised** | **-2.6** | **1.1** | **-2.32** | **.03** |
| Time 1 spatial working memory errors | -0.96 | 0.13 | -7.15 | .00 |
| Age difference | 0.02 | 0.01 | 1.58 | .12 |
| Model: *Longitudinal changes in spatial working memory errors ~ Pathlength (derived from PLV, standardised) + Age difference + time 1 spatial working memory errors*. R^2^ = .63, R^2^_adj_ = .60, F_3/32_ = 18.5, *p* < .001. | | | | |

**Supplementary discussion**

Given that zero-phase-delay synchrony is a feature of brain connectivity conserved across species (Engel et al., 1991; O’Reilly & Elsabbagh, 2021; Witham et al., 2007), we speculate that our findings can be generalised to most clinical populations. Future analyses can investigate if phase-delay distributions are altered in certain clinical phenotypes, such as dementia, given that its features include altered spike-time-dependent plasticity (Di Lorenzo et al., 2018), feature binding (Parra et al., 2010) and/or visuomotor integration (Tippett & Sergio, 2006) – functions associated with zero-phase-delay connectivity.

**Supplementary limitations**

We examined structure-function relationships with a focus on functional connectivity dependent on direct structural connections. However, while structural connectivity shapes functional connectivity, direct anatomical pathways alone leave most of the variance in the functional connectome unaccounted for (Baum et al., 2020; Finger et al., 2016; Liu et al., 2023). Previous work has accounted for this by incorporating models of information flow through the structural connectome (Finger et al., 2016). However, these models introduce varying assumptions and orders of complexity, and there is little consensus on important analytical choices in complex SC-FC modelling (Suárez et al., 2020).

In this study, our SFC-related research questions entailed a) characterising homotopic interhemispheric structural and functional connectivity and b) comparing functional connectomes produced by zero-lag inclusive versus exclusive methods to a structural connectome (and not per-say studying SFC itself). Homotopic interhemispheric functional connectivity is largely dependent on the ‘direct’ structural pathways of the corpus callosum (Engel et al., 1991; Roland et al., 2017) and direct structural connections are an established simple model to study SFC in EEG-diffusion imaging studies (Babaeeghazvini et al., 2019; Chu et al., 2015; Finger et al., 2016). Accordingly, we chose to constrain our SFC analyses to direct anatomical pathways, avoiding the additional modelling complexity and parameter space introduced by more elaborate SC–FC frameworks, in this already-complex set of analyses.

While we focused on volume conduction and signal leakage as contributors to artefactual zero-phase-delay functional connectivity, it is possible that other processes, such as residual, symmetrically distributed muscle artefact, could contribute. To our knowledge, the phase characteristics of muscle artefact in EEG have not been systematically studied, and this remains an area for future research.

We used the first principal component (PCA) of the estimated time series within each region of interest to represent its activity. While this is a common approach, other methods of dimensionality reduction exist and may lead to dissimilar adjacency matrices (Brkić et al., 2023). For example, dimensionality reduction can also be applied after computing connectivity, and is associated with higher rates of both false and true positive connections (Brkić et al., 2023) than PCA prior to computing connectivity. We used PCA prior to computing connectivity as we wanted to limit contributors of artefactual connectivity.

## Supplementary References

Babaeeghazvini, P., Rueda-Delgado, L. M., Zivari Adab, H., Gooijers, J., Swinnen, S., & Daffertshofer, A. (2019). A combined diffusion-weighted and electroencephalography study on age-related differences in connectivity in the motor network during bimanual performance. *Human Brain Mapping*, *40*(6), 1799–1813. https://doi.org/10.1002/HBM.24491

Bastos, A. M., & Schoffelen, J. M. (2016). A tutorial review of functional connectivity analysis methods and their interpretational pitfalls. *Frontiers in Systems Neuroscience*, *9*(JAN2016), 165147. https://doi.org/10.3389/FNSYS.2015.00175/BIBTEX

Baum, G. L., Cui, Z., Roalf, D. R., Ciric, R., Betzel, R. F., Larsen, B., Cieslak, M., Cook, P. A., Xia, C. H., Moore, T. M., Ruparel, K., Oathes, D. J., Alexander-Bloch, A. F., Shinohara, R. T., Raznahan, A., Gur, R. E., Gur, R. C., Bassett, D. S., & Satterthwaite, T. D. (2020). Development of structure–function coupling in human brain networks during youth. *Proceedings of the National Academy of Sciences of the United States of America*, *117*(1), 771–778. https://doi.org/10.1073/PNAS.1912034117/SUPPL_FILE/PNAS.1912034117.SAPP.PDF

Brkić, D., Sommariva, S., Schuler, A. L., Pascarella, A., Belardinelli, P., Isabella, S. L., Pino, G. Di, Zago, S., Ferrazzi, G., Rasero, J., Arcara, G., Marinazzo, D., & Pellegrino, G. (2023). The impact of ROI extraction method for MEG connectivity estimation: Practical recommendations for the study of resting state data. *NeuroImage*, *284*, 120424. https://doi.org/10.1016/J.NEUROIMAGE.2023.120424

Chu, C. J., Tanaka, N., Diaz, J., Edlow, B. L., Wu, O., Hämäläinen, M., Stufflebeam, S., Cash, S. S., & Kramer, M. A. (2015). EEG functional connectivity is partially predicted by underlying white matter connectivity. *NeuroImage*, *108*, 23–33. https://doi.org/10.1016/J.NEUROIMAGE.2014.12.033

Di Lorenzo, F., Ponzo, V., Motta, C., Bonnì, S., Picazio, S., Caltagirone, C., Bozzali, M., Martorana, A., & Koch, G. (2018). Impaired Spike Timing Dependent Cortico-Cortical Plasticity in Alzheimer’s Disease Patients. *Journal of Alzheimer’s Disease*, *66*(3), 983–991. https://doi.org/10.3233/JAD-180503/ASSET/73706CE0-5AB5-4A1E-AD3D-0638EF0B4A51/ASSETS/GRAPHIC/10.3233_JAD-180503-FIG2.JPG

Engel, A. K., König, P., Kreiter, A. K., & Singer, W. (1991). Interhemispheric Synchronization of Oscillatory Neuronal Responses in Cat Visual Cortex. *Science*, *252*(5009), 1177–1179. https://doi.org/10.1126/SCIENCE.252.5009.1177

Finger, H., Bönstrup, M., Cheng, B., Messé, A., Hilgetag, C., Thomalla, G., Gerloff, C., & König, P. (2016). Modeling of Large-Scale Functional Brain Networks Based on Structural Connectivity from DTI: Comparison with EEG Derived Phase Coupling Networks and Evaluation of Alternative Methods along the Modeling Path. *PLOS Computational Biology*, *12*(8), e1005025. https://doi.org/10.1371/JOURNAL.PCBI.1005025

Gohel, B., Lee, P., Kim, M. Y., Kim, K., & Jeong, Y. (2017). MEG Based Functional Connectivity: Application of ICA to Alleviate Signal Leakage. *IRBM*, *38*(3), 127–137. https://doi.org/10.1016/J.IRBM.2017.03.002

He, B., Astolfi, L., Valdes-Sosa, P. A., Marinazzo, D., Palva, S. O., Benar, C. G., Michel, C. M., & Koenig, T. (2019). Electrophysiological Brain Connectivity: Theory and Implementation. *IEEE Transactions on Biomedical Engineering*, *66*(7), 2115–2137. https://doi.org/10.1109/TBME.2019.2913928

Hipp, J. F., Hawellek, D. J., Corbetta, M., Siegel, M., & Engel, A. K. (2012). Large-scale cortical correlation structure of spontaneous oscillatory activity. *Nature Neuroscience 2012 15:6*, *15*(6), 884–890. https://doi.org/10.1038/nn.3101

Liu, Z. Q., Shafiei, G., Baillet, S., & Misic, B. (2023). Spatially heterogeneous structure-function coupling in haemodynamic and electromagnetic brain networks. *NeuroImage*, *278*, 120276. https://doi.org/10.1016/J.NEUROIMAGE.2023.120276

O’Reilly, C., & Elsabbagh, M. (2021). Intracranial recordings reveal ubiquitous in-phase and in-antiphase functional connectivity between homotopic brain regions in humans. *Journal of Neuroscience Research*, *99*(3), 887–897. https://doi.org/10.1002/JNR.24748

Parra, M. A., Abrahams, S., Logie, R. H., Méndez, L. G., Lopera, F., & Della Sala, S. (2010). Visual short-term memory binding deficits in familial Alzheimer’s disease. *Brain*, *133*(9), 2702–2713. https://doi.org/10.1093/BRAIN/AWQ148

Roland, J. L., Snyder, A. Z., Hacker, C. D., Mitra, A., Shimony, J. S., Limbrick, D. D., Raichle, M. E., Smyth, M. D., & Leuthardt, E. C. (2017). On the role of the corpus callosum in interhemispheric functional connectivity in humans. *Proceedings of the National Academy of Sciences of the United States of America*, *114*(50), 13278–13283. https://doi.org/10.1073/PNAS.1707050114/SUPPL_FILE/PNAS.201707050SI.PDF

Suárez, L. E., Markello, R. D., Betzel, R. F., & Misic, B. (2020). Linking Structure and Function in Macroscale Brain Networks. *Trends in Cognitive Sciences*, *24*(4), 302–315. https://doi.org/10.1016/J.TICS.2020.01.008

Tippett, W. J., & Sergio, L. E. (2006). Visuomotor integration is impaired in early stage Alzheimer’s disease. *Brain Research*, *1102*(1), 92–102. https://doi.org/10.1016/J.BRAINRES.2006.04.049

Witham, C. L., Wang, M., & Baker, S. N. (2007). Cells in somatosensory areas show synchrony with beta oscillations in monkey motor cortex. *The European Journal of Neuroscience*, *26*(9), 2677–2686. https://doi.org/10.1111/J.1460-9568.2007.05890.X
